# Supplementary material for: Identification of Crucial Residues in α-Conotoxin EI Inhibiting Muscle Nicotinic Acetylcholine Receptor
Source: Toxins (Basel). 2019 Oct 16;11(10):603. doi: 10.3390/toxins11100603 (PMC6832962; doi:10.3390/toxins11100603)
Supplement: Supplementary file 1 [file toxins-11-00603-s001.pdf]

Supplementary Materials: Identification of Crucial Residues in α-Conotoxin EI Inhibiting Muscle Nicotinic Acetylcholine Receptor

Jiong Ning, Jie Ren, Yang Xiong, Yong Wu, Manqi Zhangsun, Dongting Zhangsun, Xiaopeng Zhu and Sulan Luo

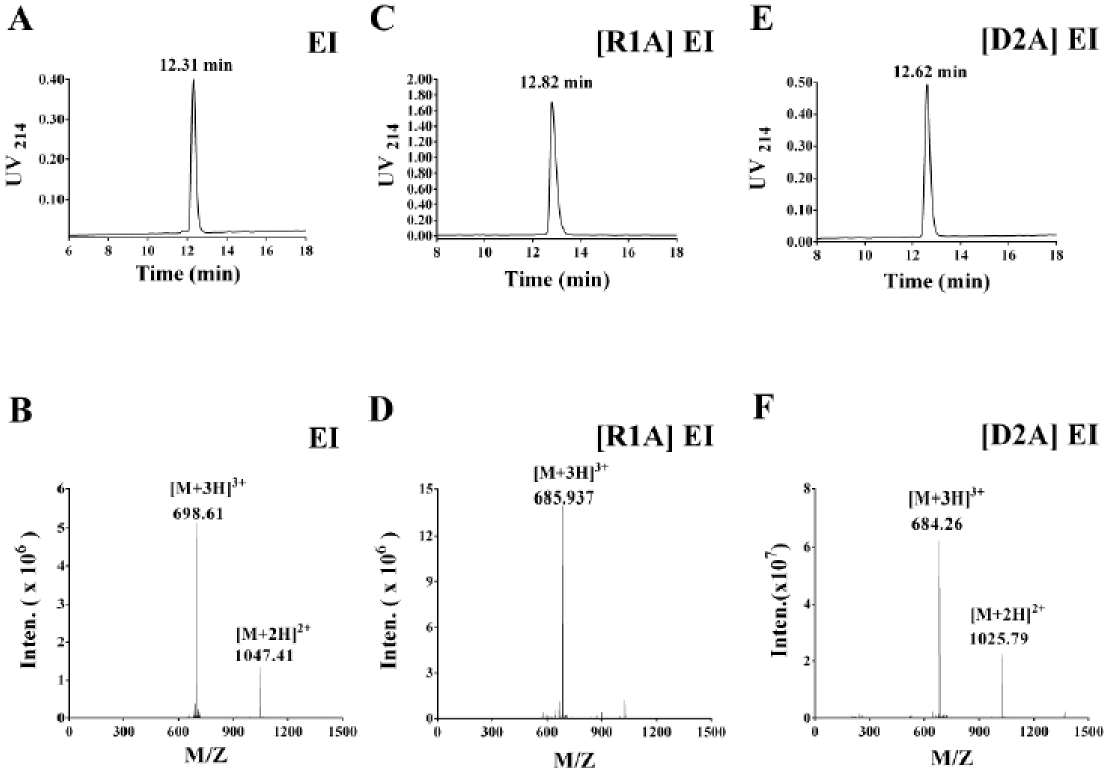

Figure S1. The HPLC chromatograms and mass spectrum of EI, [R1A] EI and [D2A] EI respectively.(**A**) HPLC chromatograms of EI and the retention of EI is 12.31 min; (**B**) electrospray ionization mass spectrometry (ESI-MS) data for EI with an observed monoisotopic mass of 2092.82 Da. (**C**) HPLC chromatograms of [R1A] EI and the retention of[R1A] EI is 12.82 min; (D) electrospray ionization mass spectrometry (ESI-MS) data for [R1A] EI with an observed monoisotopic mass of 2054.81 Da.(**E**) HPLC chromatograms of [D2A] EI and the retention of [D2A] EI is 12.62 min;(**F**) electrospray ionization mass spectrometry (ESI-MS) data for [D2A] EI with an observed monoisotopic mass of 2049.78 Da.

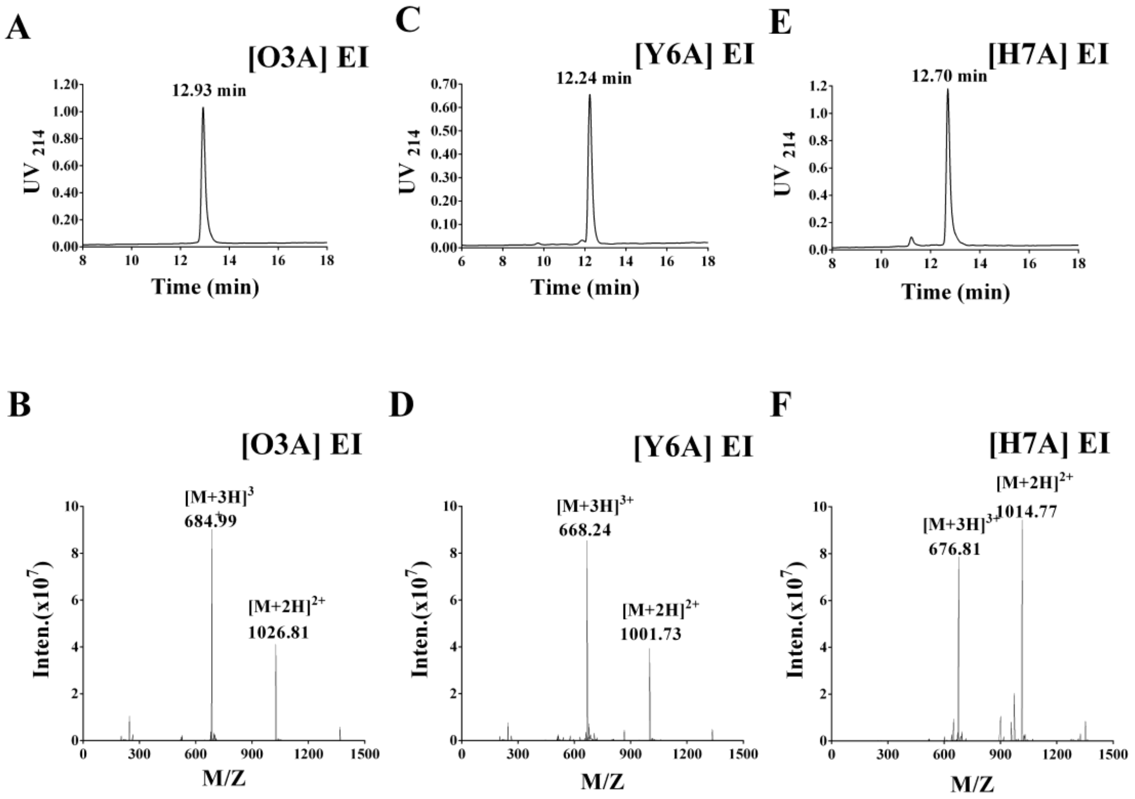

Figure S2. HPLC chromatograms and mass spectrum of [O3A] EI, [Y6A] EI and [H7A] EI respectively. (**A**) HPLC chromatograms of [O3A] EI and the retention of [O3A] EI is 12.93 min; (**B**) electrospray ionization mass spectrometry (ESI-MS) data for [O3A] EI with an observed monoisotopic mass of 2051.62 Da. (**C**)HPLC chromatograms of [Y6A] EI and the retention of [Y6A] EI is 12.24 min; (**D**) electrospray ionization mass spectrometry (ESI-MS) data for [Y6A] EI with an observed monoisotopic mass of 2051.62 Da. (E) HPLC chromatograms of GI [H7A] EI and the retention of [H7A] EI is 12.70 min; (**F**) electrospray ionization mass spectrometry (ESI-MS) data for [H7A] EI with an observed monoisotopic mass of 2027.54 Da.

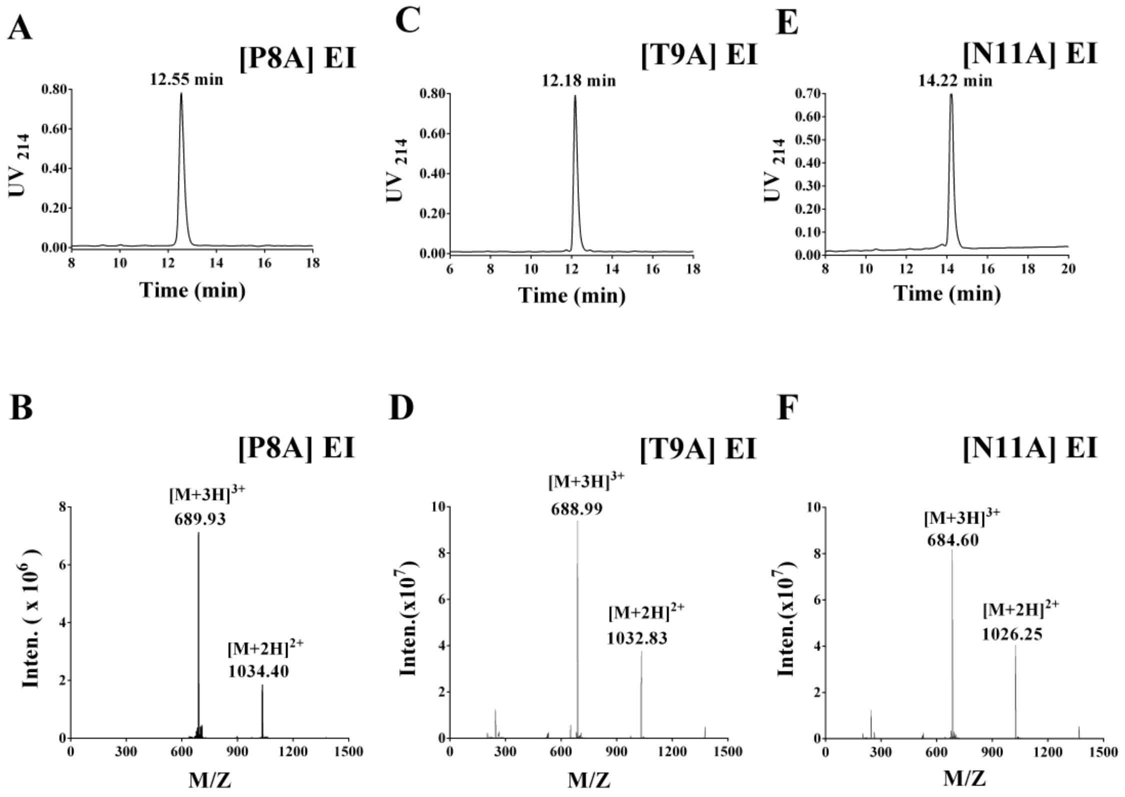

Figure S3. HPLC chromatograms and mass spectrum of [P8A] EI, [T9A] EI and [N11A] EI respectively. (**A**) HPLC chromatograms of [P8A] EI and the retention of [P8A] EI is 12.55 min; (**B**) electrospray ionization mass spectrometry (ESI-MS) data for [P8A] EIwith an observed monoisotopic mass of 2066.80 Da. (**C**) HPLC chromatograms of [T9A] EI and the retention of [T9A] EI is 12.18 min; (**D**) electrospray ionization mass spectrometry (ESI-MS) data for [T9A] EI with an observed monoisotopic mass of 2063.66 Da. (**E**) HPLC chromatograms of [N11A] EI and the retention of [N11A] EI is 14.22min; (**F**) electrospray ionization mass spectrometry (ESI-MS) data for [N11A] EI with an observed monoisotopic mass of 2050.50 Da.

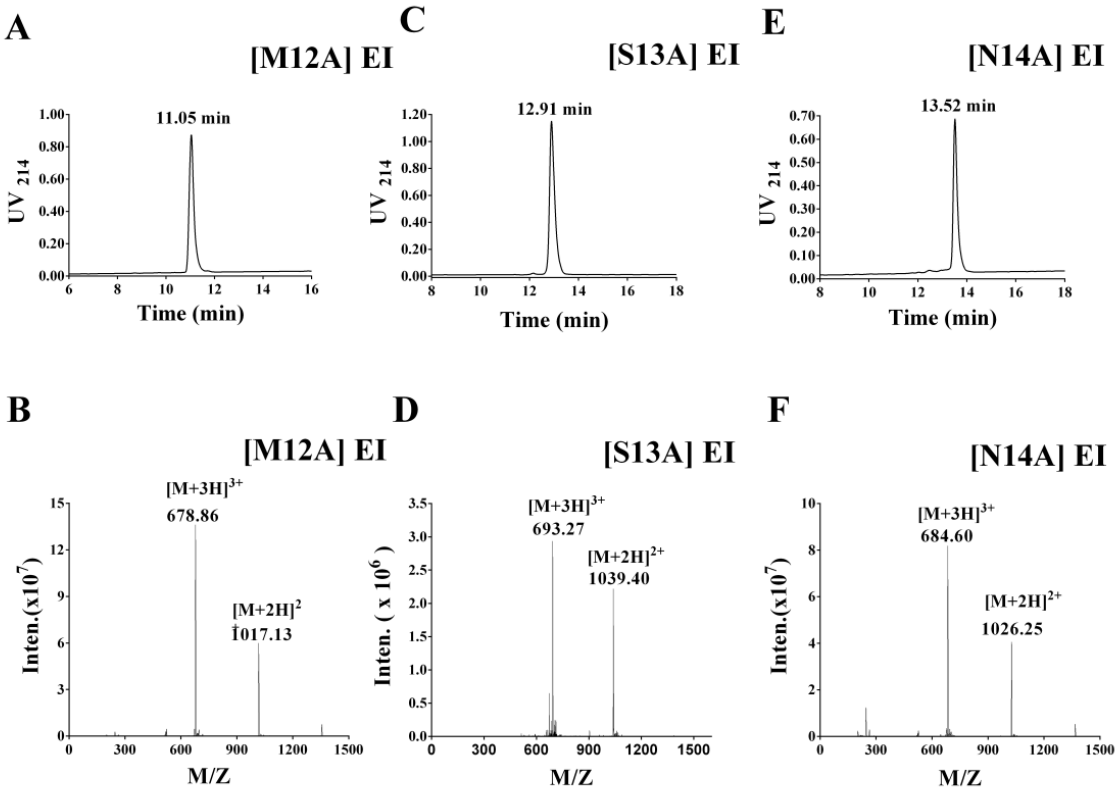

Figure S4. HPLC chromatograms and mass spectrum of [M12A] EI, [S13A] EI and [N14A] EI respectively. (**A**) HPLC chromatograms of [M12A] EI and the retention of [M12A] EI is 11.05 min; (**B**) electrospray ionization mass spectrometry (ESI-MS) data for [M12A] EI with an observed monoisotopic mass of 2033.58 Da. (**C**) HPLC chromatograms of [S13A] EI and the retention of [S13A] EI is 12.91 min; (**D**) electrospray ionization mass spectrometry (ESI-MS) data for [S13A] EI with an observed monoisotopic mass of 2076.80 Da. (**E**) HPLC chromatograms of [N14A] EI and the retention of [N14A] EI is 13.52 min; (**F**) electrospray ionization mass spectrometry (ESI-MS) data for [N14A] EI with an observed monoisotopic mass of 2050.50 Da.

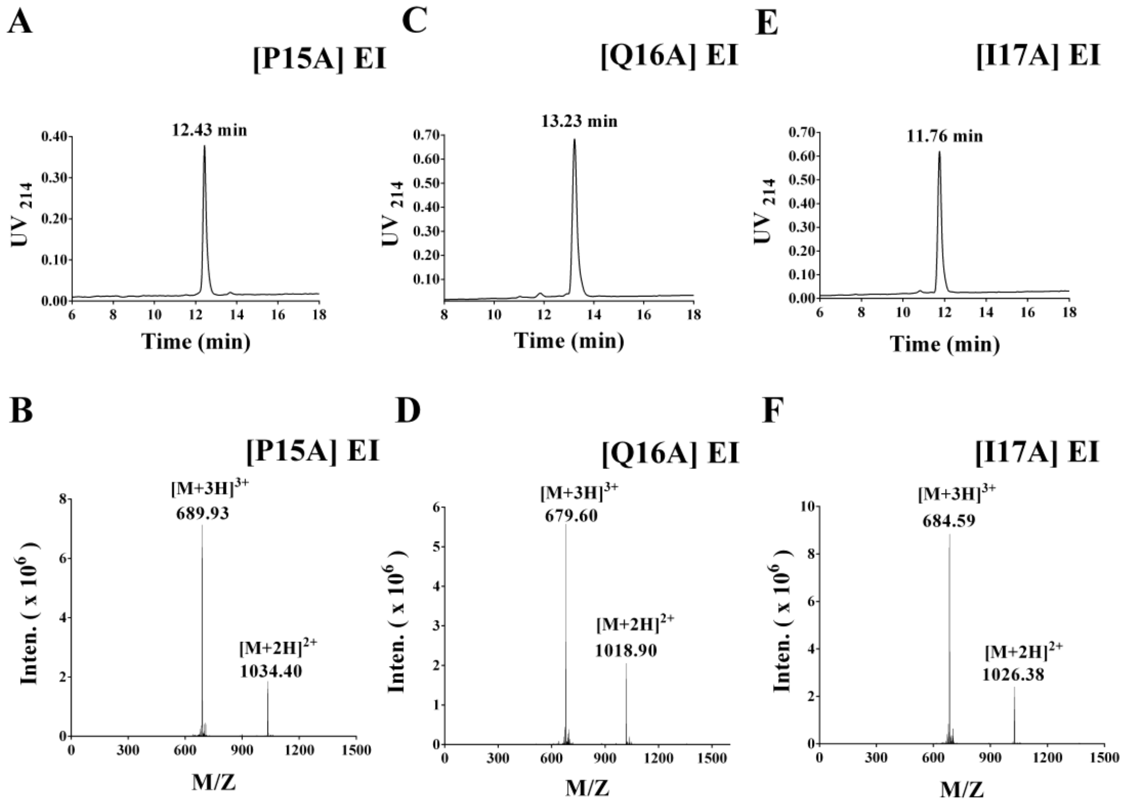

Figure S5. HPLC chromatograms and mass spectrum of [P15A] EI, [Q16A] EI and [I17A] EI respectively. (**A**) HPLC chromatograms of [P15A] EI and the retention of [P15A] EI is 12.43 min; (**B**) electrospray ionization mass spectrometry (ESI-MS) data for [P15A] EI with an observed monoisotopic mass of 2066.80 Da. (**C**) HPLC chromatograms of [Q16A] EI and the retention of [Q16A] EI is 13.23 min; (**D**) electrospray ionization mass spectrometry (ESI-MS) data for [Q16A] EI with an observed monoisotopic mass of 2035.80 Da. (**E**) HPLC chromatograms of [I17A] EI and the retention of [I17A] EI is 11.76 min; (**F**) electrospray ionization mass spectrometry (ESI-MS) data for [I17A] EI with an observed monoisotopic mass of 2050.76 Da.

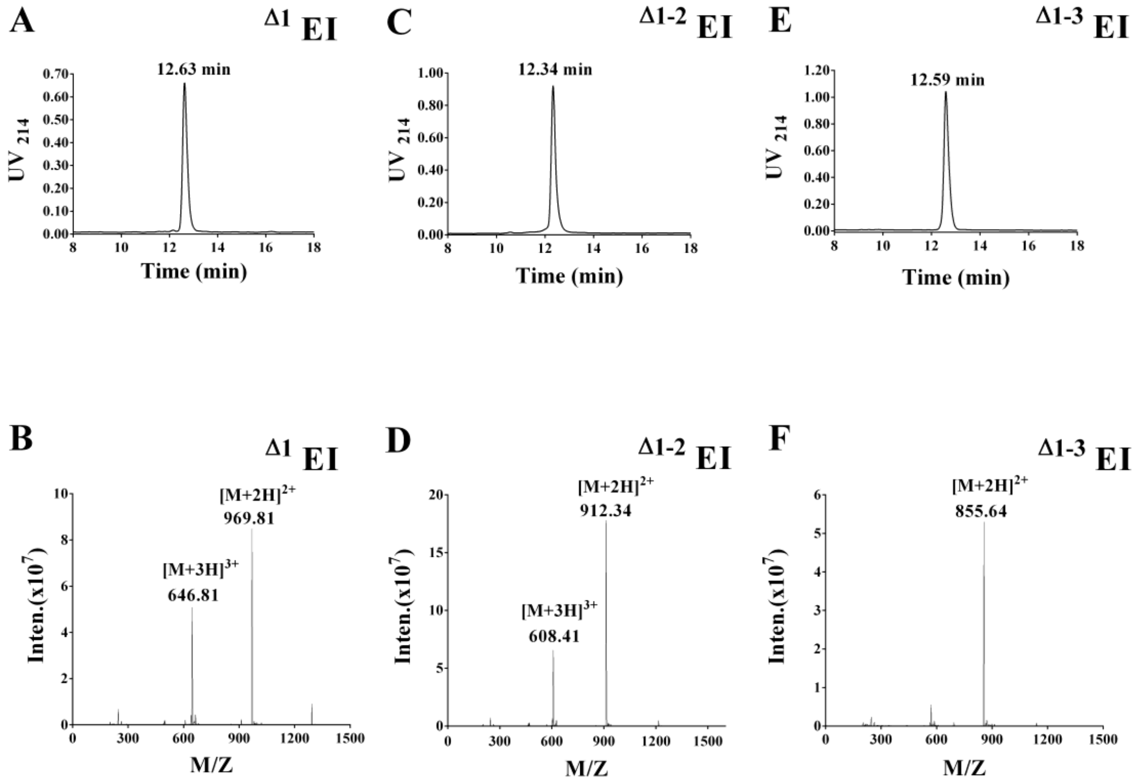

Figure S6. HPLC chromatograms and mass spectrum of $^{\triangle 1}$EI, $^{\triangle 1\text{-}2}$EI and $^{\triangle 1\text{-}3}$EI respectively. (**A**) HPLC chromatograms of $^{\triangle 1}$EIand the retention of $^{\triangle 1}$EIis 12.63 min; (**B**) electrospray ionization mass spectrometry (ESI-MS) data for $^{\triangle 1}$EIwith an observed monoisotopic mass of 1937.62 Da. (**C**) HPLC chromatograms of $^{\triangle 1\text{-}2}$EIand the retention of $^{\triangle 1\text{-}2}$EIis 12.18 min; (**D**) electrospray ionization mass spectrometry (ESI-MS) data for $^{\triangle 1\text{-}2}$EIwith an observed monoisotopic mass of 1822.68 Da. (**E**) HPLC chromatograms of $^{\triangle 1\text{-}3}$EIand the retention of $^{\triangle 1\text{-}3}$EI is 12.59 min; (**F**) electrospray ionization mass spectrometry (ESI-MS) data for $^{\triangle 1\text{-}3}$EIwith an observed monoisotopic mass of 1709.28 Da.

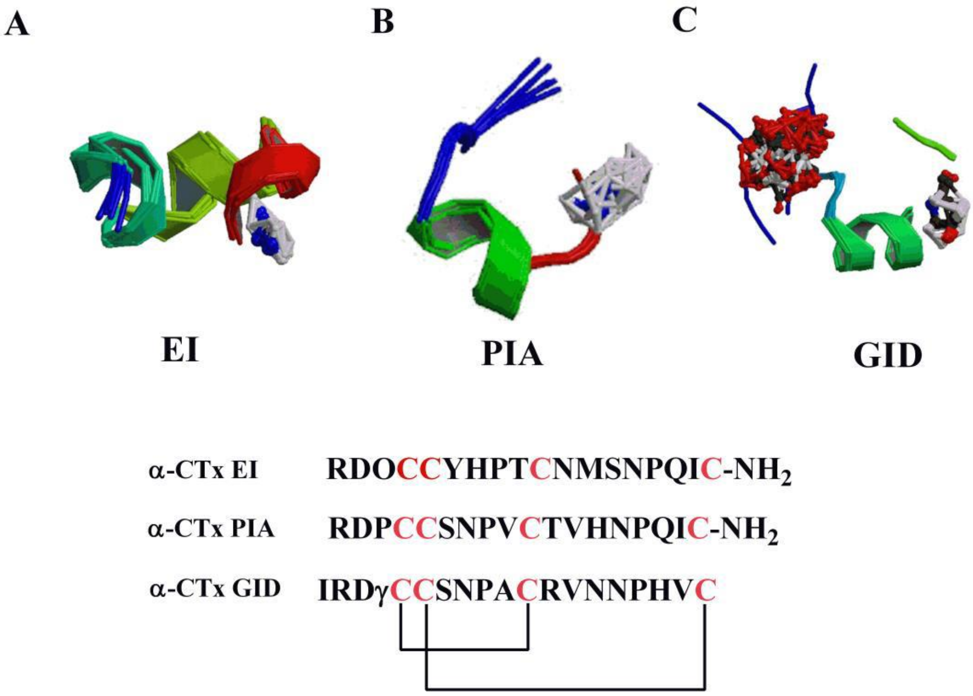

Figure S7. Three-dimensional structure of (**A**) EI (PDB: 1K64), (**B**) PIA (PDB: 1ZLC), (**C**) GID (PDB: 1MTQ), comparison of the sequences of α-Conotoxin EI (Reference 16), PIA (Reference 25) and GID (Reference 24).
